# Increased expression of the P2Y_12_ receptor is involved in the failure of autogenous arteriovenous fistula caused by stenosis

**DOI:** 10.1080/0886022X.2023.2278314

**Published:** 2023-11-23

**Authors:** Lei Liu, Jianya Gao, Yuewu Tang, Guangfeng Guo, Hua Gan

**Affiliations:** a Department of Nephrology, The First Affiliated Hospital of Chongqing Medical University, Chongqing, China; b Department of Nephrology, Chongqing University Three Gorges Hospital, Chongqing, China; c Department of Nephrology, Chongqing Three Gorges Central Hospital, Chongqing, China

**Keywords:** P2Y_12_, stenosis, arteriovenous fistula (AVF), neointima, hemodialysis

## Abstract

**Objective:**

This study investigated the role of the P2Y_12_ receptor in autogenous arteriovenous fistula (AVF) failure resulting from stenosis.

**Methods:**

Stenotic venous tissues and blood samples were obtained from patients with end-stage renal disease (ESRD) together with AVF stenosis, while venous tissues and blood samples were collected from patients with ESRD undergoing initial AVF surgery as controls. Immunohistochemistry and/or immunofluorescence techniques were utilized to assess the expression of P2Y_12_, transforming growth factor-β1 (TGF-β1), monocyte chemotactic protein 1 (MCP-1), and CD68 in the venous tissues. The expression levels of P2Y_12_, TGFβ1, and MCP-1 were quantified using quantitative reverse transcription–polymerase chain reaction and western blot analyses. Double and triple immunofluorescence staining was performed to precisely localize the cellular localization of P2Y_12_ expression.

**Results:**

Expression levels of P2Y_12_, TGFβ1, MCP-1, and CD68 were significantly higher in stenotic AVF venous tissues than in the control group tissues. Double and triple immunofluorescence staining of stenotic AVF venous tissues indicated that P2Y_12_ was predominantly expressed in α-SMA-positive vascular smooth muscle cells (VSMCs) and, to a lesser extent, in CD68-positive macrophages, with limited expression in CD31-positive endothelial cells. Moreover, a subset of macrophage-like VSMCs expressing P2Y_12_ were observed in both stenotic AVF venous tissues and control venous tissues. Additionally, a higher number of P2Y_12_^+^/TGF-β1^+^ double-positive cells were identified in stenotic AVF venous tissues than in the control group tissues.

**Conclusion:**

Increased expression of P2Y_12_ in stenotic AVF venous tissues of patients with ESRD suggests its potential involvement in the pathogenesis of venous stenosis within AVFs.

## Introduction

Arteriovenous fistula (AVF) is the preferred vascular access for patients with end-stage renal disease (ESRD) undergoing hemodialysis. Nonetheless, the AVF’s initial-year patency rate rests at just 60%, with a subsequent 35% AVF failure rate after 2 years [1,2]. This elevated failure rate escalates patient morbidity and mortality, as patients must resort to non-AVF dialysis access [1–3]. Moreover, primary AVF failure necessitates endovascular or surgical interventions to salvage the AVF, thereby subjecting patients to risks and burdens while causing healthcare costs to surge by up to fourfold [4]. Despite the prevailing acknowledgment of the imperative for heightened primary AVF patency rates, the dearth of effective therapies for vascular access dysfunction underscores the formidable underlying challenges [5]. The principal cause of AVF dysfunction stems from neointima formation and venous neointimal hyperplasia (VNH), marked by an abundance of smooth muscle cells (SMCs), myofibroblasts, fibroblasts, and macrophages, which culminate in venous stenosis and foster thrombosis [6–9]. VNH pathology encompasses multiple vascular biology pathways, including inflammation, uremia, hypoxia, shear stress, and augmented thrombogenicity. These mechanisms are believed to synergistically operate through interlinked cytokine cascades and potentially epigenetic changes, ultimately yielding adverse remodeling and fistula failure [8–10]. However, the underlying pathophysiology underlying AVF occlusion remains nebulous, and no pharmacological remedies are extant to forestall VNH/venous stenosis development. Consequently, delving into the molecular and cellular mechanisms underpinning VNH is imperative to tackle this quandary.

The P2Y_12_ receptor, a member of the P2 receptor family, was originally discerned in platelets. Activation through adenosine diphosphate (ADP) triggers platelet aggregation and thrombosis, making the P2Y_12_ receptor on platelets the cardinal target of antiplatelet drugs. Notwithstanding initial assertions that the P2Y_12_ receptor was confined to platelets and specific brain regions [11], subsequent investigations have unveiled its presence in microglial cells [12,13], dendritic cells [14], oligodendrocytes [15], oligodendrocyte precursor cells [16], astrocytes [17], endothelial cells (ECs) [18,19], vascular SMCs (VSMCs) [20–23], osteoclasts [24], monocytes [25,26], macrophages [26–28], and leukocytes [29].

Corroborating recent research, the P2Y_12_ receptor finds expression in VSMCs and participates in regulating their physiological and pathological functions, thereby advancing the atherogenic process [23,30,31]. Furthermore, elevated P2Y_12_ receptor levels have been observed in vascular injuries such as coronary artery disease and carotid artery disease. Activation of the P2Y_12_ receptor propels VSMC proliferation and migration, whereas inhibition of the receptor mitigates atherosclerosis [23,31]. These findings suggest that the P2Y_12_ receptor is implicated in atherosclerosis progression through VSMCs. Antagonizing the P2Y_12_ receptor curtails platelet-leukocyte aggregate formation, diminishes proinflammatory cytokine levels, and hampers VSMC migration and proliferation. This endows P2Y_12_ receptor inhibitors with added anti-inflammatory and anti-atherosclerosis benefits beyond their antithrombotic effects, thus designating the P2Y_12_ receptor as a potential novel therapeutic target for atherosclerosis [32,33].

At present, the expression and function of the P2Y_12_ receptor within the vascular wall of cephalic veins and stenotic venous segments in patients with ESRD and AVF have not been explored. It is therefore imperative to scrutinize the expression and plausible role of the P2Y_12_ receptor in the vascular wall of cephalic veins and stenotic AVF venous segments in patients with ESRD.

In the present study, vascular tissues (cephalic veins) obtained from patients undergoing their initial AVF surgery, along with stenotic vascular tissues excised during AVF repair for stenosis cases, were collected to evaluate the expression of P2Y_12_ and related genes. The objective was to determine the expression of P2Y_12_ and related genes within vascular tissues and to investigate the role of the P2Y_12_ receptor in the failure of autogenous AVFs caused by stenosis.

## Materials and methods

### Study subjects

Between June 2021 and December 2022, eligible patients with ESRD receiving treatment at the Department of Nephrology, Chongqing University Three Gorges Hospital, were recruited for the current study. The experimental group (EG) consisted of 15 patients (10 males and 5 females) who met the inclusion criteria. Additionally, 16 patients with ESRD who underwent autogenous AVF surgery for the first time were selected and included in the control group (CG; 9 males and 7 females).

The Ethics Committee of Chongqing University Three Gorges Hospital granted approval for the study (No. 2021-82, Chinese Medical Research Filing No. MR-50-23-026776). All patients provided written consent to participate, and the research adhered to the principles outlined in the Declaration of Helsinki (2013).

The inclusion criteria for the experimental group included cumulatively: Patients meeting the criteria for AVF Clinical Maturation [34]; individuals with a normally functioning autogenous AVF for more than 3 months and a dialysis duration of fewer than 10 years; AVF created through an end-to-side anastomosis between the radial artery and the cephalic vein of the forearm; patients with venous stenosis near the anastomosis leading to insufficient blood flow during hemodialysis; meeting the criteria for AVF stenosis [34,35] as determined by a vascular access specialist, necessitating fistula repair.

The inclusion criteria for the control group included: Patients with ESRD requiring long-term renal replacement therapy through blood purification and those evaluated by a vascular access specialist as needing an autogenous AVF anastomosis.

The exclusion criteria were as follows: (1) patients younger than 18 years or older than 75 years; (2) patients with peripheral vascular thrombosis or stenosis due to arteriovenous malformations or atrial fibrillation; (3) patients with diabetes, malignant tumors, severe infections, severe liver diseases, rheumatic diseases, or other serious systemic conditions; (4) patients who received hormonal or immunosuppressant treatment within 6 months before tissue collection; (5) pregnant individuals.

For the experimental group, venous stenosis segment tissues removed during AVF repair were collected from 15 patients. In the control group, discarded venous tissues (cephalic veins) from 16 patients during the initial autogenous AVF anastomosis were collected. A portion of the collected tissues was fixed with 4% paraformaldehyde for 24–48 h to prepare paraffin sections, while the remaining tissue was immediately stored at −80 °C for total RNA and protein extraction.

Clinical characteristics, including age, sex, and serum biochemistry data, were documented and analyzed by the researchers involved in the study.

### Reagents

Anti-P2Y_12_ rabbit polyclonal antibody (NBP1-78249) and anti-TGFβ1 rabbit polyclonal antibody (NBP1-80289) were purchased from Novus Bio (USA). Anti-β-Actin (13E5) rabbit monoclonal antibody (4970S) was purchased from Cell Signaling Technologies (USA). Anti-MCP1 rabbit polyclonal antibody (HA500042) was purchased from Hua Bio (China). Anti-CD68 rabbit polyclonal antibody (GB113150), anti-CD31 rabbit polyclonal antibody (GB11063-2), and anti-alpha smooth muscle actin (α-SMA) mouse monoclonal antibody (GB13044) were purchased from Service Bio (China). CY3-Tyramide(G1223), iF488-Tyramide (G1231), iF647-Tyramide (G1232), and FITC-Tyramide (G1222) were purchased from Service Bio (China). DAB Immunohistochemistry Color Development Kit (G1212) was purchased from Service Bio (China). TRIZOL (G3013) reagent was purchased from Service Bio (China). Oligonucleotide primers for PCR were custom synthesized by BGI Genomics Co., Ltd (China). cDNA Synthesis Kit (G592) and qPCR reaction Kit (G891) were purchased from Applied Biological Materials (ABM) Inc (CAN).

### Histological evaluation and immunohistochemistry

The collected tissue samples fixed with 4% paraformaldehyde were longitudinally embedded in paraffin and sectioned into 4-μm-thin contiguous sections. These sections were subjected to staining with hematoxylin and eosin (HE) and Elastic-Van Gieson (EVG) to assess histological changes in the venous tissue.

For immunohistochemistry (IHC), the paraffin sections were deparaffinized using xylene and gradually dehydrated with gradient alcohol. Antigen retrieval was achieved using a 0.01 mol/L citrate buffer solution, and endogenous peroxidase was blocked with 3% hydrogen peroxide. Following natural cooling to room temperature, the sections were washed with 0.1 mol/L phosphate-buffered saline and blocked with 3% bovine serum albumin (BSA). Subsequently, the sections were incubated overnight at 4 °C with primary antibodies targeting P2Y_12_ (1:100), TGF-β1 (1:200), MCP-1 (1:200), and CD68 (1:500), respectively. Afterward, horseradish peroxidase-labeled goat anti-rabbit secondary antibodies were applied. Protein visualization was performed at room temperature using a DAB Immunohistochemistry Color Development Kit. Image J software was utilized to measure the optical density of the positive expression of P2Y_12_, TGF-β1, MCP-1, and CD68. Each sample was evaluated in five fields of view, and the outcomes were presented as the mean ± standard deviation. Pearson’s correlation coefficients were employed for the experimental group data to ascertain the relationships between the IHC expression of P2Y_12_ and those of TGF-β1, MCP-1, or CD68. Negative controls were included in the staining performed with the IgG control.

### Immunofluorescence assay

Deparaffinized paraffin-embedded sections underwent antigen retrieval in an EDTA buffer (pH 8.0) by sub-boiling temperature for 8 min, followed by two standing periods of 8 min and another sub-boiling temperature for 7 min for antigen retrieval. The sections were then blocked with 3% BSA and incubated overnight at 4 °C with antibodies against P2Y_12_ (1:1000 or 3000), TGF-β1 (1:200), α-SMA (1:500 or 1000), CD68 (1:200), and CD31 (1:200). After incubation, the sections were treated with appropriate secondary antibodies for 1 h at room temperature, followed by staining with the corresponding tyramide signal amplification dyeing working solution for 10 min at room temperature. Finally, the sections were counterstained with 4′,6-diamidino-2-phenylindole (DAPI) to label cellular nuclei, and then subjected to spontaneous fluorescence quenching and mounting. The positively stained cells were quantified per high-power field. Negative controls underwent staining with IgG. Images were captured using fluorescent microscopy.

### Western blot

Total protein was extracted using protein lysate, and the protein was quantified by the BCA method. After adding protein loading buffer and boiling for denaturation for 10 min, 10 μg of denatured protein was separated by 12% SDS-polyacrylamide gel electrophoresis and transferred to polyvinylidene fluoride membrane by wet transfer method. After blocking with defatted milk for 1 h at room temperature, the membrane was incubated overnight at 4 °C in a primary antibody solution containing P2Y_12_ (1:500), TGF-β1 (1:1000), MCP-1 (1:500), and β-Actin (1:1000) in a refrigerator shaker. Membranes were washed thrice with tris buffered saline with tween, each time for 10 min, and then incubated with secondary antibodies for 1 h at room temperature. The enhanced chemiluminescence reagent was added, and the membrane was exposed to the FluorChem M imaging system. The results were analyzed using ImageJ software, and the ratio of the gray value of the target protein band to the gray value of the internal reference β-actin band was used as the relative expression of the target protein.

### Quantitative reverse transcriptase–polymerase chain reaction (qRT-PCR)

Total RNAs were extracted from tissues with TRIZOL reagent. From each sample, one microgram of RNA was reversed-transcribed to cDNA using the cDNA Synthesis Kit. cDNAs were diluted and used for the template for RT-qPCR assay, following the manufacturer’s instructions. GAPDH was used as an internal reference gene for normalization. All gene expression was quantified relative to GAPDH expression using the 2-^ΔΔCt^ method. Primer sequences for the target genes were as follows: P2Y12-F: 5′-ATGCCAAACTGGGAACAGGA-3′, P2Y12-R: 5′-AAATGGCCTGGTGGTCTTCT-3′, TGF-β1-F: 5′-GGAAATTGAGGGCTTTCGCC-3′, TGF-β1-R: 5′-CCGGTAGTGAACCCGTTGAT-3′, MCP-1-F: 5′-GAAAGTCTCTGCCGCCCTT-3′, MCP-1-R: 5′-GGTGACTGGGGCATTGATTG-3′, GAPDH-F: 5′-GCACCGTCAAGGCTGAGAAC-3′, GAPDH -R: 5′-TGGTGAAGACGCCAGTGGA-3′.

### Statistical analysis

Statistical analysis was conducted using SPSS 27.0 and GraphPad Prism 9 software. Clinical characteristic data were presented as mean ± standard deviation (SD), while other experimental quantitative data were represented as mean ± standard error of the mean (SEM). All experiments were repeated at least three times. Two-group comparisons were analyzed using t-tests. Pearson’s correlation coefficients were applied to assess associations between IHC expression of P2Y_12_ and those of TGF-β1, MCP-1, or CD68. *P* values less than 0.05 were considered statistically significant.

## Results

### Clinical characteristics of the patients

In this study, no significant differences were observed between the two groups in terms of age, sex, leukocyte count, neutrophil percentage, hemoglobin levels, platelet count, total protein, albumin, urea, creatinine, calcium, phosphorus, potassium, alkaline phosphatase (ALP), intact parathyroid hormone (iPTH), C-reactive protein (CRP), and β2-microglobulin (β2-MG; Table 1).

**Table 1.** Comparison of the clinical data between the two groups of patients.

Variable	Control group	Experimental group	*p* value
Number of patients, *n*	16	15	/
Age (years)	58.44 ± 14.48	54.20 ± 12.76	0.3956
Sex (male: female)	9:7	10:5	0.7160
Leukocyte (×10^9^/L)	6.260 ± 1.547	5.878 ± 1.934	0.5471
N (%)	69.09 ± 9.004	71.99 ± 9.439	0.3886
Hemoglobin (g/L)	95.06 ± 8.442	107.9 ± 25.96	0.0713
Platelet (×10^9^/L)	170.8 ± 55.13	145.9 ± 48.74	0.1935
Total protein (g/L)	66.31 ± 7.290	64.21 ± 11.80	0.5530
Albumin (g/L)	39.92 ± 3.754	41.83 ± 4.983	0.2348
Urea (mmol/L)	26.85 ± 7.931	24.77 ± 9.198	0.5042
Creatinine (μmol/L)	769.7 ± 136.5	898.4 ± 321.7	0.5010
Calcium (mmol/L)	2.141 ± 0.1695	2.248 ± 0.1891	0.1083
Phosphorus (mmol/L)	1.771 ± 0.4791	2.117 ± 0.7211	0.1248
Potassium (mmol/L)	4.432 ± 0.7887	4.608 ± 0.7527	0.5303
ALP (U/L)	84.50 ± 35.75	84.36 ± 38.18	0.9767
iPTH (pg/mL)	400.2 ± 247.5	546.0 ± 390.2	0.3846
CRP (mg/L)	5.763 ± 8.373	10.54 ± 10.55	0.0670
β2 -MG (mg/L)	16.51 ± 5.665	20.49 ± 5.283	0.0735

The measures were presented as mean ± standard deviation, *p* > 0.05 was considered not statistically significant. N: neutrophil percentage; iPTH: intact parathyroid hormone; ALP: alkaline phosphatase; CRP: c-reactive protein; β2-MG: β2-Microglobulin.

### Expression of P2Y_12_, TGF-β1, MCP-1, and CD68 is upregulated in stenotic AVF venous tissues

**Figure 1.**
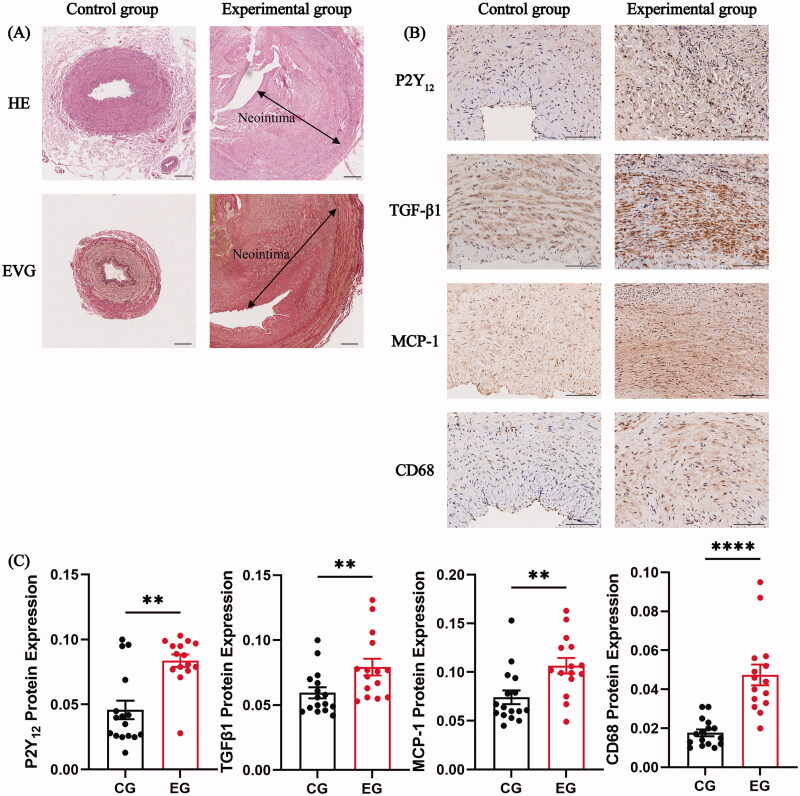
Hematoxylin-eosin (HE) and Elastic-Van Gieson (EVG) staining, and immunohistochemical staining for P2Y_12_, TGF-β1, MCP-1, and CD68 in the venous tissues between the two groups. (A) Comparison of HE and EVG staining results in venous tissues between the two groups. Scale bar: 200 μm. (B and C) Immunohistochemical staining for P2Y_12_, TGF-β1, MCP-1, and CD68 in the venous tissues of both groups. Representative images of P2Y_12_, TGF-β1, MCP-1, and CD68 staining are presented (B). semiquantitative analysis results revealed a significant increase in the expression of P2Y_12_, TGF-β1, MCP-1, and CD68 in the experimental group as compared to that in the control group (C). two-sample t-tests were performed for statistical analysis (C). Each bar represents mean ± standard error of the mean (SEM). significant differences are denoted as **p < 0.01 and ****p < 0.0001. Scale bar: 100 μm. CG and EG refer to the control and experimental groups, respectively.

To investigate the role of P2Y_12_ in chronic kidney disease (CKD)-related AVF failure, we initially assessed the expression of P2Y_12_ and related genes such as TGF-β1, MCP-1, and CD68 in the stenotic venous tissues of the experimental group and the cephalic veins of the control group. Histological staining using HE and EVG revealed significant thickening of the intima in the blood vessels of the experimental group, leading to pronounced stenosis and near occlusion of the lumen. By contrast, the control group displayed no conspicuous intimal thickening (Figure 1A). IHC demonstrated positive expression of P2Y_12_, TGF-β1, MCP-1, and CD68 in vascular tissues, indicated by brown or yellow-brown staining. The results clearly indicated a marked increase in the expression of P2Y_12_, TGF-β1, MCP-1, and CD68 in stenotic venous tissues of the experimental group compared with the venous tissues of the control group (Figure 1B, C).

Furthermore, we conducted a correlational analysis to explore the relationship between P2Y_12_ expression and that of TGF-β1, MCP-1, and CD68 in the stenotic venous tissues of the experimental group. The data revealed positive correlations between P2Y_12_ and the expression of TGF-β1 (*r* = 0.7211; *p* = 0.0032), MCP-1 (*r* = 0.7621; *p* = 0.0010), and CD68 (*r* = 0.6961; *p* = 0.0039).

**Figure 2.**
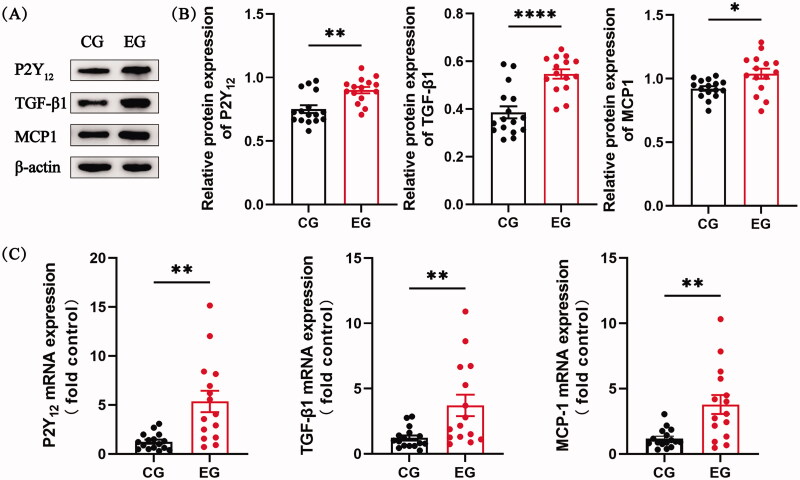
The expression of P2Y_12_, TGF-β1, and MCP-1 was assessed by western blot and qRT-PCR. (A and B) The results showed that the expression of P2Y_12_, TGF-β1, and MCP-1 was significantly increased in stenotic venous tissues of the experimental group as compared to that in venous tissues of the control group. The quantitation of the expression ratio was determined by western blot (shown with representative images) and quantified *via* densitometry analysis. (C) qRT-PCR analysis of P2Y_12_, TGF-β1, and MCP-1 expression in venous tissues of the control and experimental groups. The results showed that a significant increase in the expression of P2Y_12_, TGF-β1, and MCP-1 in stenotic venous tissues of the experimental group as compared to that in venous tissues of the control group. Two-sample t-test were performed for statistical analysis (B and C). Each bar represents mean ± SEM. Significant differences are denoted as *p < 0.05, **p < 0.01, and ****p < 0.0001. CG and EG refer to the control and experimental groups respectively.

Subsequently, we proceeded to analyze the expression of P2Y_12_, TGF-β1, and MCP-1 using western blot and qRT-PCR. Figures 2A, B depict the western blot results, demonstrating the expression levels of P2Y_12_, TGF-β1, and MCP-1 in vascular tissues. Considerable increases in the protein expression levels of P2Y_12_, TGF-β1, and MCP-1 were evident in the experimental group when compared with the control group. Figure 2C illustrates the qRT-PCR results, indicating the expression levels of P2Y_12_, TGF-β1, and MCP-1 in vascular tissues. The experimental group exhibited significantly higher expression levels of P2Y_12_, TGF-β1, and MCP-1 compared with the control group (Figure 2C).

### Distribution of the P2Y_12_ receptor

**Figure 3.**
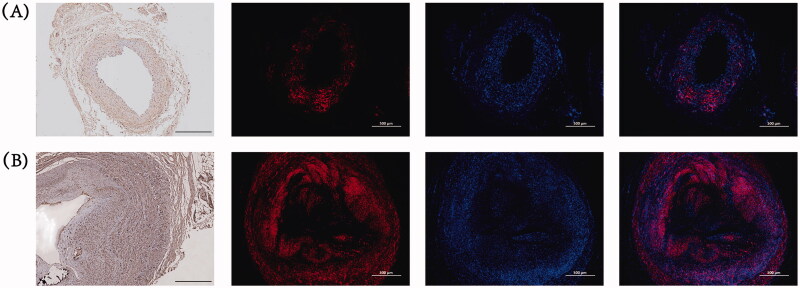
Immunohistochemical and immunofluorescent staining for P2Y_12_ in venous tissues from the both groups. The representative images of P2Y_12_ staining in the control group (A) and experimental group (B). Scale bar: 500 μm.

For a more comprehensive understanding of the role of P2Y_12_ in AVF stenosis development, we delved into the distribution of P2Y_12_ in the cephalic veins of the control group and the stenotic venous tissues of the experimental group. Our findings showed modest expression of P2Y_12_ in the intima, media, and adventitia of the cephalic veins in the control group, with more pronounced expression within the media (Figure 3A). In the stenotic venous tissues of the experimental group, P2Y_12_ expression was increased in the neointima, media, and adventitia, with the neointima and media constituting the primary sites of expression (Figure 3B).

### Cellular localization of P2Y_12_ receptor expression

Given the observed upregulation of P2Y_12_ in stenotic venous tissues during CKD-related AVF failure, a double immunofluorescent staining experiment was conducted to elucidate the cellular localization of P2Y_12_ expression. Macrophages were labeled with CD68 (green), ECs with CD31 (green), and VSMCs with α-SMA (green), while co-staining was performed with P2Y_12_ (red).

**Figure 4.**
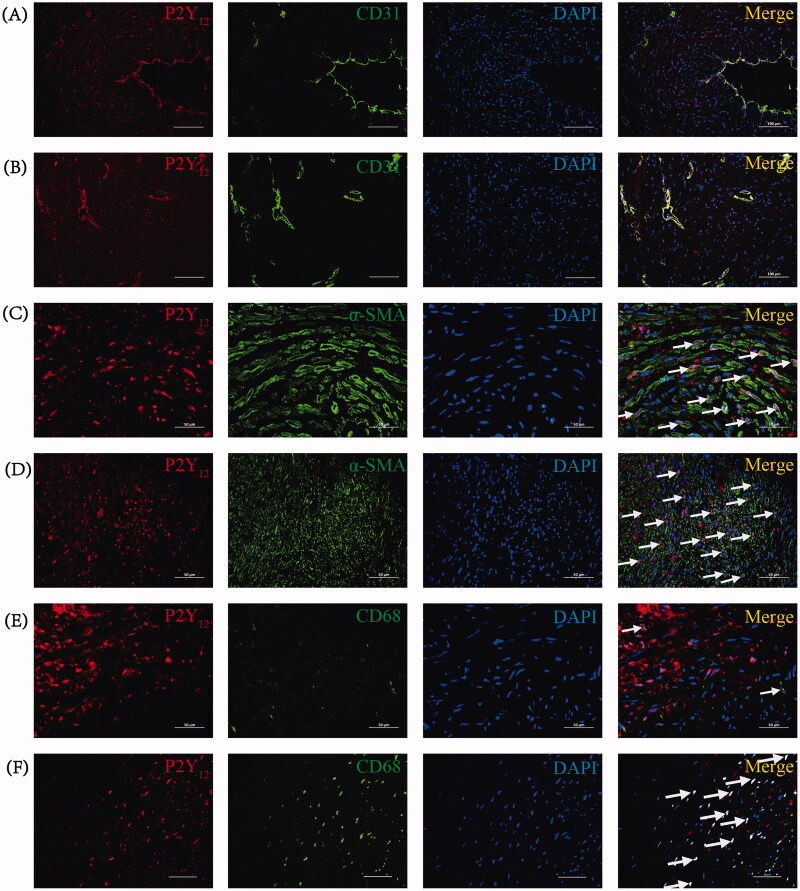
Double immunofluorescent staining for the cellular localization of P2Y_12_ expression in venous tissues between the two groups. (A) Representative photomicrographs showing P2Y_12_ (red) and CD31 (green) in venous tissues of the control group. Scale bar: 100 μm. (B) Representative photomicrographs showing P2Y_12_ (red) and CD31 (green) in stenotic venous tissues of the experimental group. Scale bar: 100 μm. (C) Representative photomicrographs showing P2Y_12_ (red) and α-SMA (green) in venous tissues of the control group. White arrows point to P2Y_12_^+^/α-SMA^+^ cells. Scale bar: 50 μm. (D) Representative photomicrographs showing P2Y_12_ (red) and α-SMA (green) in stenotic venous tissues of the experimental group. White arrows point to P2Y_12_^+^/α-SMA^+^ cells. Scale bar: 50 μm. (E) Representative photomicrographs showing P2Y_12_ (red) and CD68 (green) in venous tissues of the control group. White arrows point to P2Y_12_^+^/CD68^+^ cells. Scale bar: 50 μm. (F) Representative photomicrographs showing P2Y_12_ (red) and CD68 (green) in stenotic venous tissues of the experimental group. White arrows point to P2Y_12_^+^/CD68^+^ cells. Scale bar: 50 μm.

Co-staining CD31 with P2Y_12_ unveiled P2Y_12_ expression within ECs in both the experimental and control groups. In the control group, P2Y_12_ was evident in CD31^+^ ECs within the intima of venous tissues (Figure 4A). In the experimental group, notable intimal hyperplasia and substantial damage to intimal integrity led to sporadic P2Y_12_ expression in proliferative intima-associated ECs (Figure 4B). Furthermore, neovascular ECs within the neointima also exhibited P2Y_12_ expression (Figure 4B).

By co-staining α-SMA with P2Y_12_ in venous tissues of both the control and experimental groups, we observed that the majority of P2Y_12_-expressing cells also concurrently expressed α-SMA. Within the control group’s cephalic veins, P2Y_12_ was predominantly present in α-SMA^+^ VSMCs within the media (Figure 4C). Conversely, within the stenotic venous tissues of the experimental group, P2Y_12_ expression was concentrated in α-SMA^+^ VSMCs within the neointima and media (Figure 4D). Significantly more P2Y_12_^+^/α-SMA^+^ double-positive cells were detected in stenotic AVF venous tissues (Figure 7A).

In the control group, only a few CD68^+^ cells were evident within cephalic veins, accompanied by a minor presence of P2Y_12_^+^/CD68^+^ double-positive cells (Figure 4E). Conversely, the experimental group displayed a higher incidence of P2Y_12_^+^/CD68^+^ double-positive cells within stenotic AVF venous tissues (Figures 4F and 7B).

Cumulatively, the aforementioned investigations in stenotic AVF venous tissues indicated that P2Y_12_ expression was primarily confined to α-SMA^+^ VSMCs and present in CD68^+^ macrophages, with limited presence in CD31^+^ ECs. This implies that VSMCs and macrophages, acting as inflammatory cells, may contribute to the advancement of AVF stenosis through P2Y_12_ receptors, which are likely pivotal in AVF stenosis formation.

### Macrophage-like VSMCs exist in cephalic veins and stenotic AVF venous tissues of patients with ESRD

The pronounced role of VSMCs in the development of atherosclerotic plaques is well established. Notably, the existence of macrophage-like VSMCs has been identified within human atherosclerotic plaques, hinting at their involvement in atherosclerosis progression [36–40]. However, the presence of such macrophage-like VSMCs within the cephalic veins and stenotic AVF venous tissues of patients with ESRD has not been reported.

**Figure 5.**
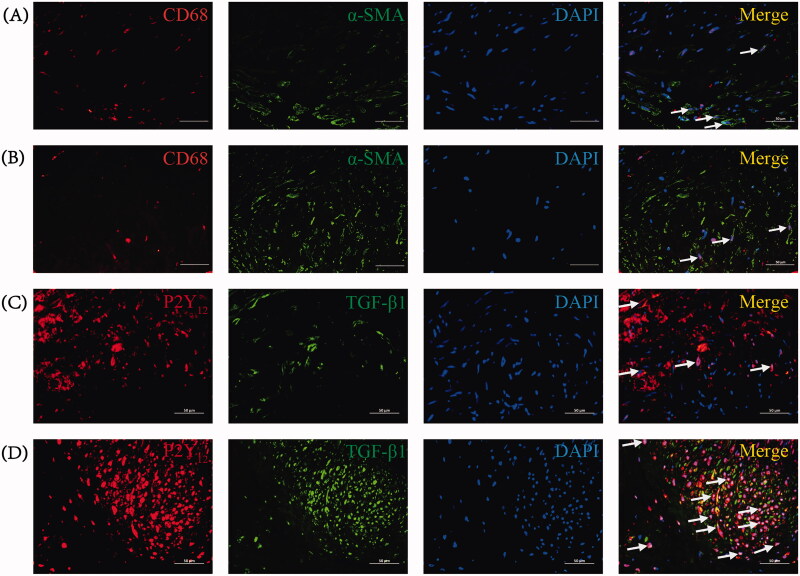
Double immunofluorescent staining for the CD68 with α-SMA or P2Y_12_ with TGF-β1 in venous tissues between the two groups. (A) Representative photomicrographs showing CD68(red) and α-SMA (green) in venous tissues of the control group. White arrows point to CD68^+^/α-SMA^+^ cells. Scale bar: 50 μm. (B) Representative photomicrographs showing CD68(red) and α-SMA (green) in stenotic venous tissues of the experimental group. White arrows point to CD68^+^/α-SMA^+^ cells. Scale bar: 50 μm. (C) Representative photomicrographs showing P2Y_12_(red) and TGF-β1(green) in venous tissues of the control group. White arrows point to P2Y_12_^+^/TGF-β1^+^ cells. Scale bar: 50 μm. (D) Representative photomicrographs showing P2Y_12_(red) and TGF-β1(green) in stenotic venous tissues of the experimental group. White arrows point to P2Y_12_^+^/TGF-β1^+^ cells. Scale bar: 50 μm.

In this study, we confirmed the presence of a small number of CD68^+^/α-SMA^+^ double-positive cells in the cephalic veins of the control group by performing double staining for CD68 with α-SMA (Figure 5A). This finding established the presence of macrophage-like VSMCs within the cephalic veins of the control group. However, only a limited number of CD68^+^/α-SMA^+^ double-positive cells, indicative of macrophage-like VSMCs, were detected in the stenotic venous tissues of the experimental group (Figure 5B). Notably, the majority of P2Y_12_^+^/CD68^+^ double-positive cells in stenotic AVF venous tissues were identified as monocyte-derived macrophages.

**Figure 6.**
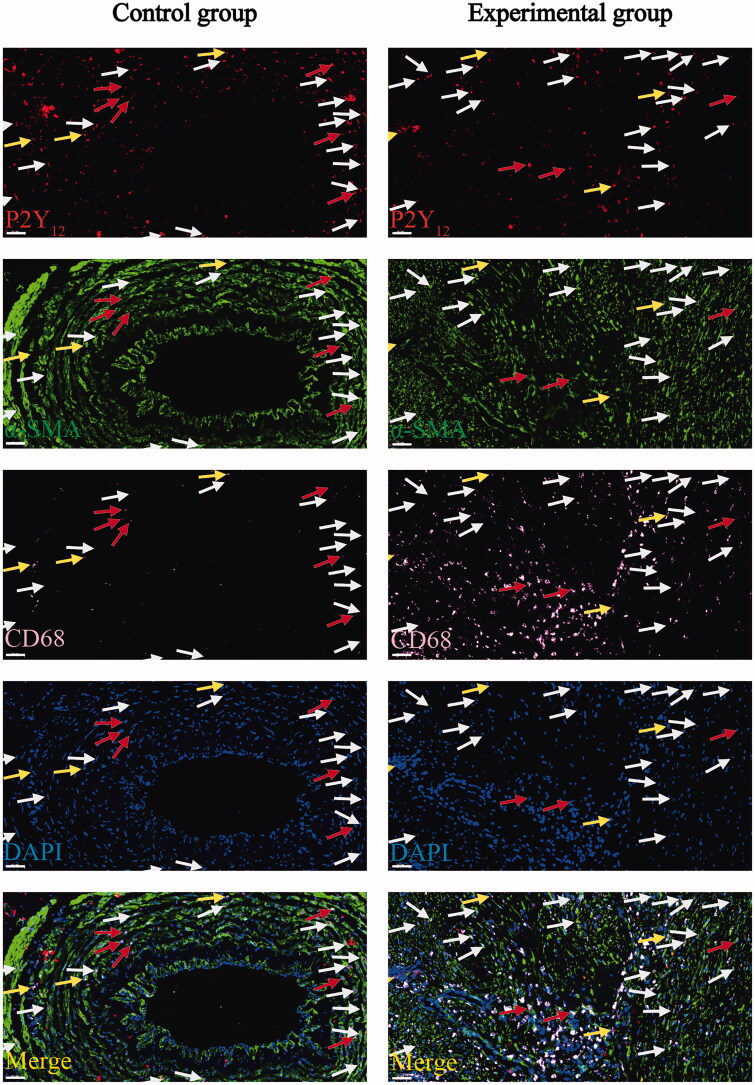
Triple immunofluorescent staining for the P2Y_12_ (red), α-SMA (green), and CD68 (pink) in venous tissues between the two groups. Representative photomicrographs showing P2Y_12_ (red), α-SMA (green), and CD68 (pink) in venous tissues of the control group and experimental group. Red arrows point to P2Y_12_^+^/α-SMA^+^/CD68^+^ cells, white arrows point to P2Y_12_^+^/α-SMA^+^/CD68^-^ cells, yellow arrows point to P2Y_12_^+^/α-SMA^-^/CD68^+^ cells. Scale bar: 50 μm.

To validate the above observations, we conducted triple immunofluorescent staining experiments employing P2Y_12_ (red), α-SMA (green), and CD68 (pink). This analysis revealed a minor population of P2Y_12_^+^/α-SMA^+^/CD68^+^ triple-positive cells in both the cephalic veins of the control group and the stenotic venous tissues of the experimental group (Figure 6). These triple-positive cells indicated the presence of macrophage-like VSMCs expressing P2Y_12_. Intriguingly, all macrophage-like VSMCs (CD68^+^/α-SMA^+^ cells) displayed P2Y_12_ expression. However, P2Y_12_^+^/α-SMA^+^ double-positive cells did not invariably express CD68, suggesting that P2Y_12_ could be expressed in both macrophage-like VSMCs (P2Y_12_^+^/CD68^+^/α-SMA^+^ cells) and non-macrophage-like VSMCs (P2Y_12_^+^/CD68^-^/α-SMA^+^ cells). Furthermore, the existence of P2Y_12_^+^/CD68^+^/α-SMA^-^ cells indicated that certain P2Y_12_ receptors were expressed in CD68^+^ monocyte-derived macrophages, rather than originating from macrophage-like VSMCs (Figure 6). However, the precise role of macrophage-like VSMCs in the formation of AVF stenosis remains elusive and necessitates further experimental investigation, particularly through dynamic research in mouse AVF models.

**Figure 7.**
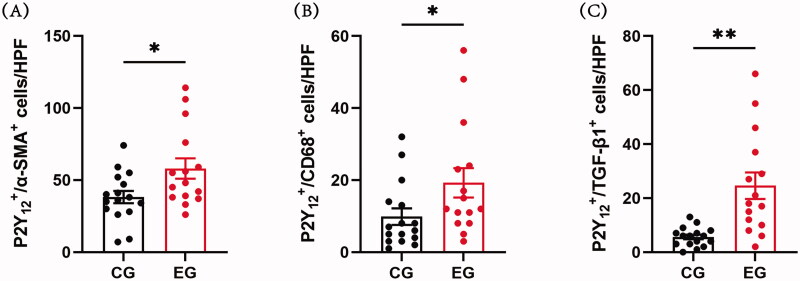
The statistical analysis of P2Y_12_^+^ and α-SMA^+^ or CD68^+^ or TGF-β1^+^ double-positive cells comparison in venous tissues between the two groups. (A) Bar graph shows quantification of P2Y_12_^+^/α-SMA + cells in venous tissues of the control group and experimental group. (B) Bar graph shows quantification of P2Y_12_^+^/CD68^+^ cells in venous tissues of the control group and experimental group. (C) Bar graph shows quantification of P2Y_12_^+^/TGF-β1^+^ cells in venous tissues of the control group and experimental group. Each bar represents mean ± SEM. Significant differences are denoted as *p < 0.05 and **p < 0.01. CG and EG refer to the control and experimental groups respectively.

### P2Y_12_^+^/TGF-β1^+^ double-positive cells are upregulated in stenotic AVF venous tissues

Considering that TGF-β1 has been established as a critical player in AVF stenosis formation [41–43], we proceeded to delve into the relationship between P2Y_12_ and TGF-β1. Correlation analysis using immunohistochemistry data demonstrated a positive correlation between P2Y_12_ and TGF-β1 expression (*r* = 0.7211; *p* = 0.0032). Consequently, we performed double staining for P2Y_12_ and TGF-β1, revealing an elevated presence of P2Y_12_^+^/TGF-β1^+^ double-positive cells in the stenotic venous tissues of the experimental group when compared with the control group (Figures 5C,D, and 7C). This suggests a potential joint involvement of P2Y_12_ and TGF-β1 in the progression of AVF stenosis formation, warranting further investigation into the specific underlying mechanisms.

## Discussion

In this investigation, we noted a conspicuous rise in the expression of the P2Y_12_ receptor within stenotic AVF venous tissues, primarily localized within the neointimal and medial layers of the venous tissues. This observation is novel and supports our initial hypothesis that increased P2Y_12_ receptor expression might contribute to the development of AVF stenosis. Evidently, the P2Y_12_ receptor could play a pivotal role in neointima formation and AVF stenosis progression.

Furthermore, our study unveiled the predominant presence of the P2Y_12_ receptor in α-SMA-positive VSMCs found in both the media and neointima. The enigma surrounding the neointimal cell, encompassing its origin, functions, and molecular regulation, makes it a focal point in vascular biology. Given that VSMCs constitute the principal inhabitants of the vascular wall, their contribution to the neointimal cell population in AVFs is almost certain. Substantial experimental data corroborate the involvement of venous and arterial SMCs in the AVF intima [44]. Notably, the customary origin of proliferating cells in neointimal hyperplasia (NH) is the vascular media housing SMCs. Impelled by endothelial and smooth muscle cell injury resulting from hemodynamic stress and mechanical trauma, SMCs migration from the media to the intima transpires. Once in the intima, SMCs undergo proliferation and differentiate into a secretory phenotype (myofibroblasts) [45]. Mature SMCs are pivotal for maintaining equilibrium between medial wall thickening (facilitated by the proliferation of differentiated SMCs) and neointimal hyperplasia (stemming from dedifferentiated SMCs that induce NH and failure) [46].

Our investigation documented a significant increase in P2Y_12_^+^/α-SMA^+^ double-positive cells within stenotic AVF venous tissues. This elevation in P2Y_12_ and α-SMA double-positive cell expression prompted us to speculate their potential involvement in neointimal growth. This insinuates that α-SMA-positive VSMCs might actively partake in AVF stenosis progression through P2Y_12_ receptors, thereby underscoring the plausible significance of P2Y_12_ receptors in AVF stenosis formation.

Numerous studies have already demonstrated that P2Y_12_ receptors possess the capacity to amplify VSMC proliferation and migration [22,23,47,48], consequently exerting a noteworthy role in atherogenesis. We hypothesize that the augmented expression of P2Y_12_ receptors within VSMCs in stenotic AVF venous tissues could similarly propel VSMC proliferation and migration, thereby fostering neointimal growth. However, the validation of this hypothesis necessitates further exploration through animal AVF models.

Reports indicate that up to 50% of macrophage-like cells within atherosclerotic plaques stem from SMCs [49]. The question looms whether macrophage-like VSMCs function akin to monocyte-derived macrophage subsets in atherosclerosis [38]. Overall, the presence of macrophage markers in VSMCs does not imply full functionality as macrophages or cell specificity, making it difficult to predict their impact on atherosclerotic plaque growth and stability. Nevertheless, based on their pro-inflammatory profile, macrophage-like VSMCs are likely to be detrimental to plaque stability [50].

Within our study, macrophage-like VSMCs were discerned in both stenotic AVF venous tissues and control venous tissues. However, their presence within stenotic AVF venous tissues was limited. Despite this observation, the specific role of these macrophage-like VSMCs in AVF stenosis formation remains enigmatic, necessitating further experimental investigation, particularly dynamic studies employing mouse AVF models.

The emergence of venous stenosis subsequent to AVF surgery closely intertwines with inflammation [9,51,52]. Excessive inflammation underpins AVF failure, and curtailing proinflammatory stimuli can mitigate VNH and venous stenosis in AVF [53,54]. Studies involving ApoE^−/−^ mice demonstrated that genetic ablation of the P2Y_12_ gene led to reduced monocyte/macrophage infiltration within atherosclerotic lesions [55]. Likewise, research by Satonaka et al. established that ADP, through the VSMC P2Y_12_ receptor, elicits vascular inflammatory changes by upregulating MCP-1 and promoting monocyte adhesion. Furthermore, the P2Y_12_ receptor plays a role in modulating crucial inflammatory mediators such as MCP-1[56].

Our current study revealed a significant elevation in the expression of CD68 and MCP-1 within stenotic AVF venous tissues, indicating a pronounced inflammatory response within AVF stenosis veins. Additionally, we identified a notable surge in the expression of P2Y_12_^+^/CD68^+^ double-positive cells within stenotic AVF venous tissues, although the presence of macrophage-like VSMCs was minimal. This suggests that the majority of P2Y_12_^+^/CD68^+^ double-positive cells within stenotic AVF venous tissues emanated from monocyte-macrophage sources. Additionally, the identification of P2Y_12_^+^/CD68^+^/α-SMA^−^ cells substantiates that some P2Y_12_ receptors are expressed in CD68^+^ monocyte-derived macrophages, rather than originating from macrophage-like VSMCs. We conjecture that this increase in expression could be linked to an escalated local inflammatory response within stenotic AVF venous tissues. This discovery implies that macrophages, as key players in inflammation, potentially contribute to the progression of AVF stenosis through P2Y_12_ receptors.

TGF-β, a multifunctional cytokine, exerts control over extracellular matrix (ECM) deposition and SMC proliferation, both of which hold pivotal roles in neointimal hyperplasia leading to AVF stenosis [42,43,57–64]. Elevated TGF-β expression within stenoses in human AVF [43], and genetic polymorphisms that express high amounts of TGF-β protein correlate with lower AVF patency [65]. Martinez et al. [66] demonstrated that postoperative venous fibrosis increases universally in patients, and only postoperative venous medial fibrosis determines the stenotic potential of intimal hyperplasia in AVF, ultimately establishing vascular fibrosis as a hallmark of AVF failure. TGF-β assumes a central role in kidney and vascular fibrosis and stands as a determinant for AVF functioning [67]. Additionally, TGF-β orchestrates inflammation within the remodeling AVF wall [42]. The pivotal role of inflammation in vascular remodeling and AVF maturation, with macrophages playing an essential part in AVF maturation, underscores the significance of inflammatory cells [68–71]. Together, this evidence suggests that excessive TGF-β signaling underpins AVF stenosis, positioning the TGF-β signaling pathway as a prospective therapeutic target for preventing AVF failure.

Within our study, TGF-β1 expression within stenotic AVF venous tissues significantly surpassed that in the control group, aligning with previous research [46]. Furthermore, we noted a substantial increase in the expression of P2Y_12_^+^/TGF-β1^+^ double-positive cells within stenotic AVF venous tissues. This leads us to speculate that P2Y_12_ receptors might partake in AVF stenosis formation through the TGF-β1 signaling pathway, although this hypothesis necessitates further investigation.

Considering that P2Y_12_ receptors are expressed across various cell types within the AVF wall, each potentially influencing AVF stenosis progression, we posit that P2Y_12_ receptors could activate the TGF-β1 pathway within the AVF wall and participate in the excessive inflammation, promoting VSMC proliferation, migration, and ultimately, the evolution of AVF stenosis.

Nonetheless, our study does bear limitations. It did not encompass animal models or cellular assays, precluding the exploration of the effects of P2Y_12_ receptor inhibitor application in stenosis prevention. Additionally, the study’s small sample size and the absence of non-stenotic vascular tissues from AVF as controls limited its scope. In subsequent investigations, expanding the sample size and including patients with diabetes mellitus as research subjects will enhance the study’s robustness.

In conclusion, our study divulges an elevated presence of P2Y_12_ receptors in stenotic AVFs, which contributes to neointimal hyperplasia and stenosis progression. The surfeit expression of P2Y_12_ receptors within stenotic AVF venous tissues strongly correlates with AVF failure. Consequently, P2Y_12_ receptor blockade holds promise as a therapeutic strategy to mitigate stenosis progression in AVFs. The P2Y_12_ receptors could emerge as pivotal targets in averting and managing AVF failure.

## Supplementary Material

Supplemental Material

Supplemental Material

Supplemental Material

Supplemental Material

## Data Availability

The data used to support the findings of this study are available from the corresponding author upon request.
