# Supplementary figures and images for: Increased expression of the P2Y12 receptor is involved in the failure of autogenous arteriovenous fistula caused by stenosis
Source: Ren Fail. 2023 Nov 23;45(2):2278314. doi: 10.1080/0886022X.2023.2278314 (PMC11073481; doi:10.1080/0886022X.2023.2278314)

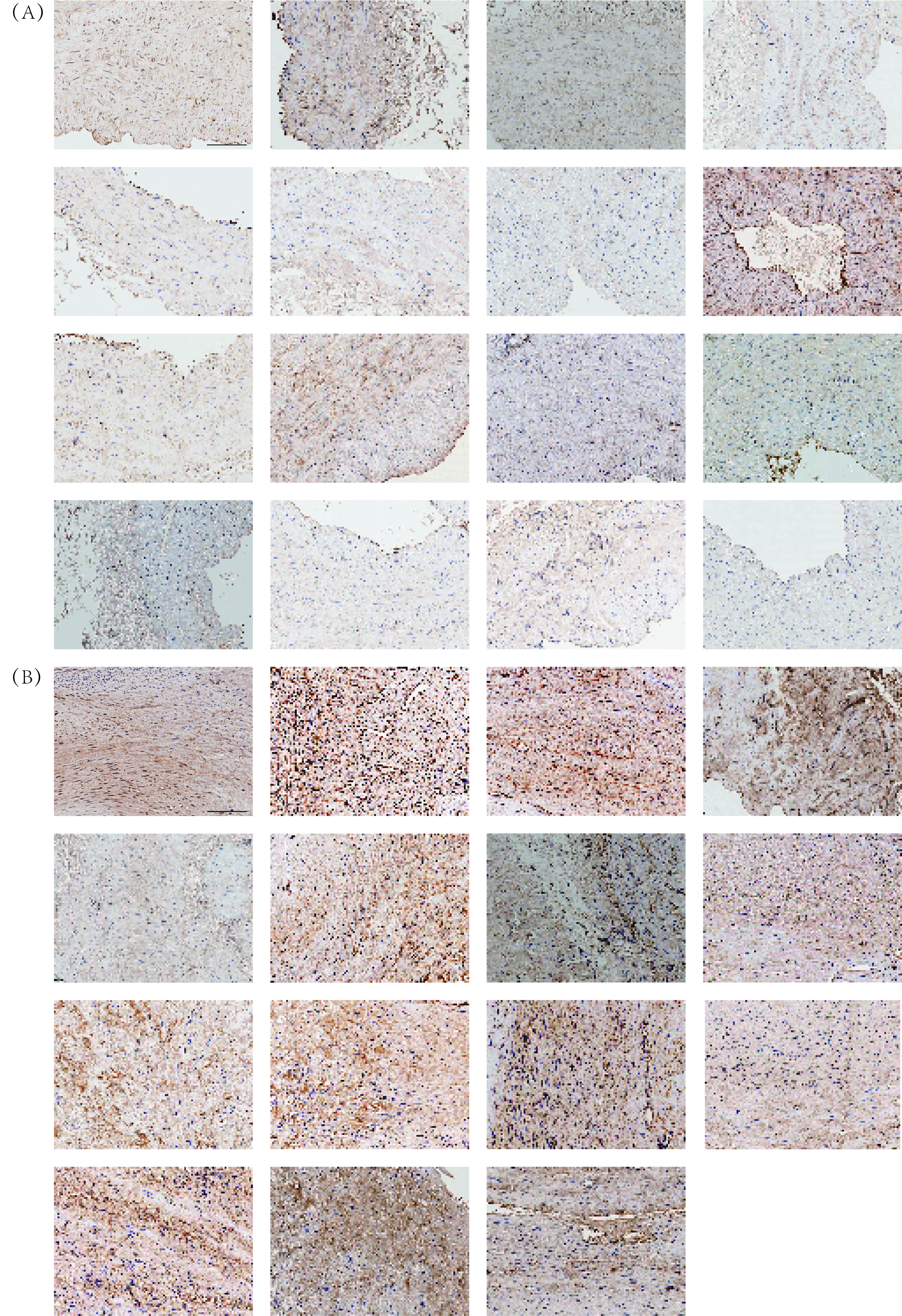

Supplement: Supplemental Material [file IRNF_A_2278314_SM2885.tif]

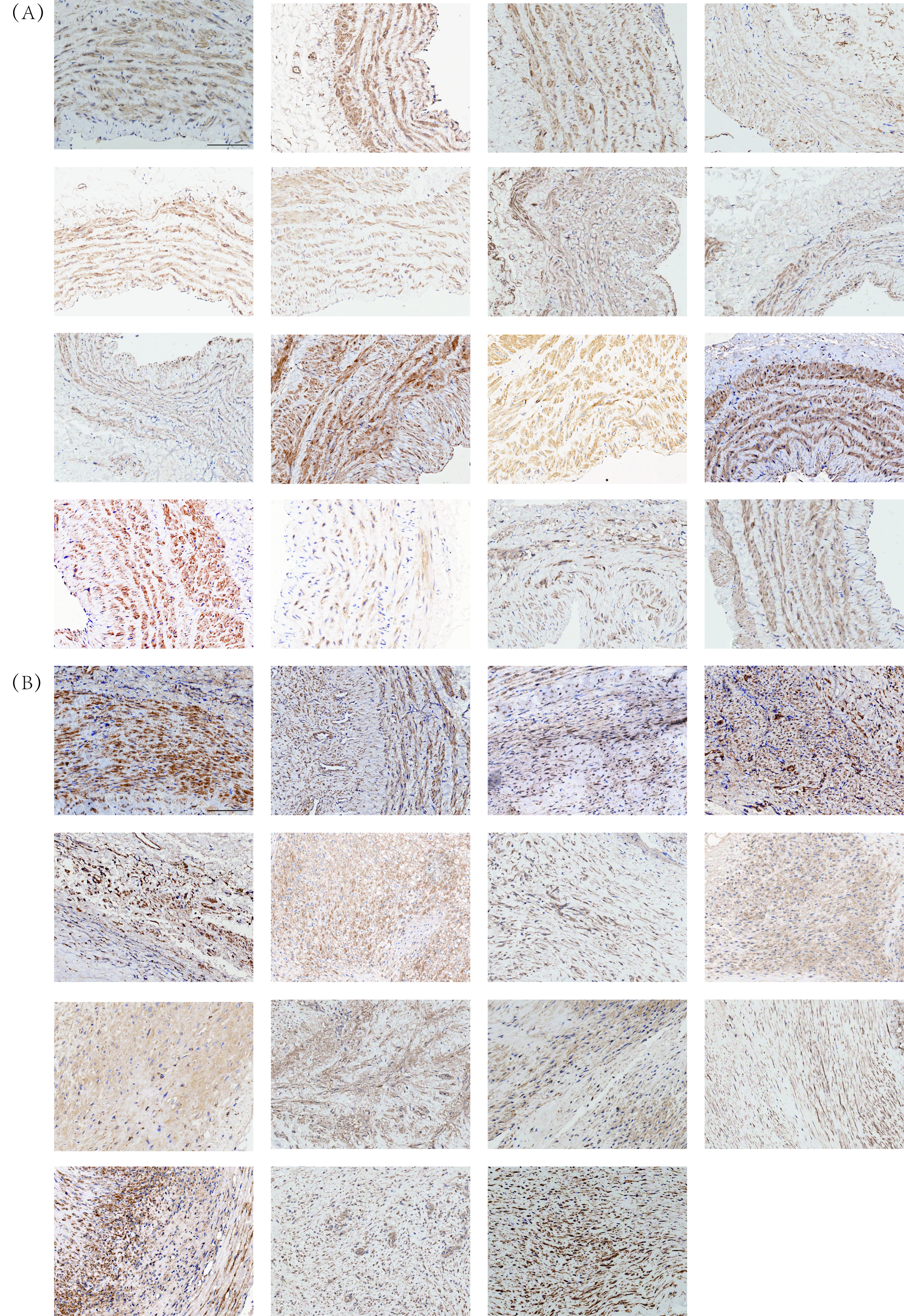

Supplement: Supplemental Material [file IRNF_A_2278314_SM2871.tif]

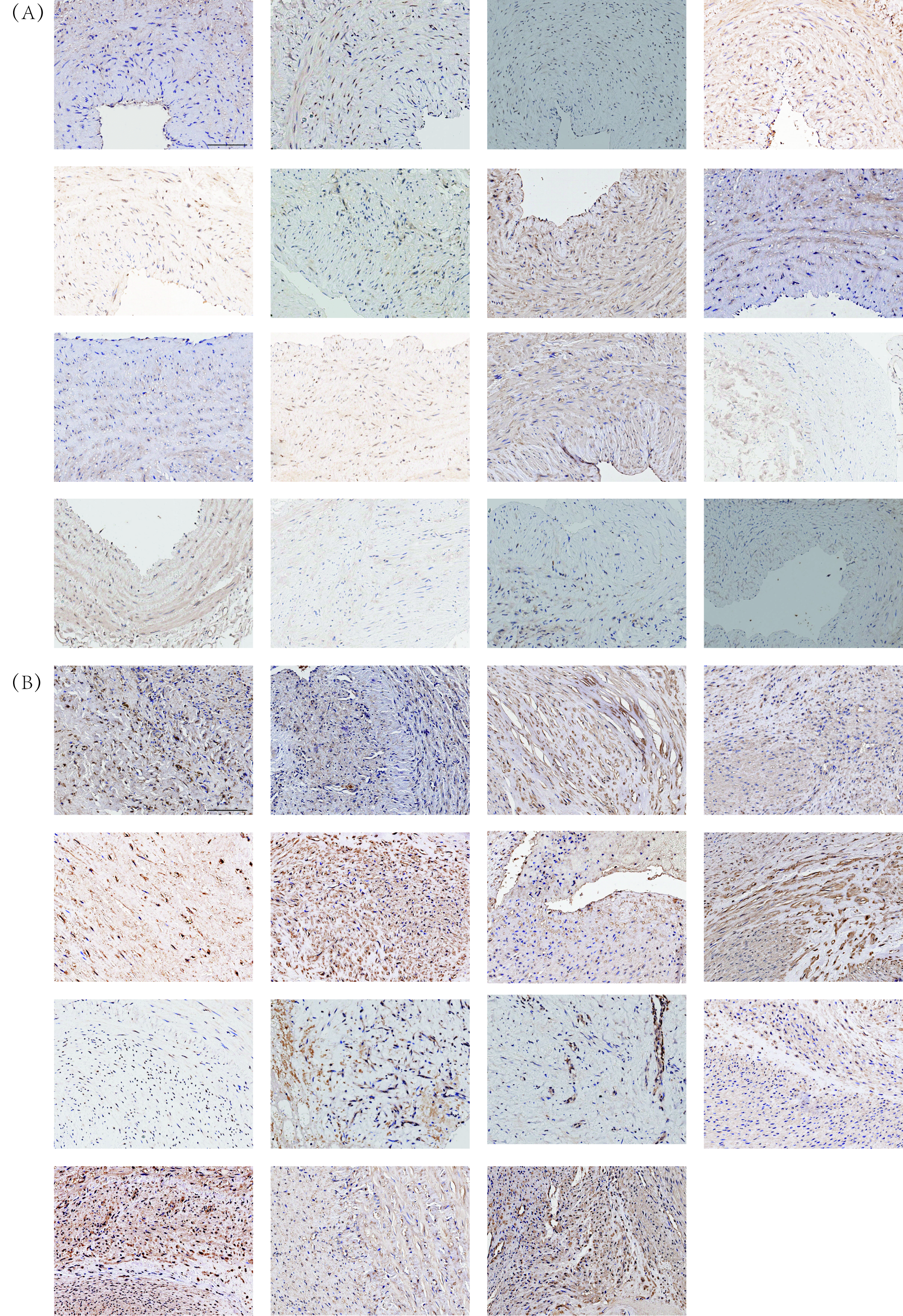

Supplement: Supplemental Material [file IRNF_A_2278314_SM2864.tif]
